# Length of Postoperative Hospital Stay and Related Factors After Lobectomy for Lung Cancer: A Pre-enhanced Recovery After Surgery (ERAS) Single Center Assessment

**DOI:** 10.7759/cureus.54724

**Published:** 2024-02-22

**Authors:** Ho Tat Bang, Tran Thanh Vy, Nguyen Van Tap

**Affiliations:** 1 Thoracic and Vascular Department, University Medical Center Ho Chi Minh City, University of Medicine and Pharmacy at Ho Chi Minh City, Ho Chi Minh, VNM; 2 Health Organization and Management Department, Faculty of Public Health, University of Medicine and Pharmacy at Ho Chi Minh City, Ho Chi Minh, VNM; 3 Cardiovascular and Thoracic Surgery Department, Faculty of Medicine, University of Medicine and Pharmacy at Ho Chi Minh City, Ho Chi Minh, VNM; 4 Faculty of Medical Management, Nguyen Tat Thanh University, Ho Chi Minh City, VNM

**Keywords:** vietnam, lung cancer, lobectomy, eras, complications, length of hospital stay

## Abstract

Background: Lobectomy for lung cancer often presents a lot of potentially severe complications after surgery for patients. Enhanced Recovery After Surgery (ERAS) is a program to improve unexpected events. When implementing ERAS, there needs to be evidence of relevant factors that prolong hospital stays to encourage the participation of medical staff and leaders. This study is to determine the length of hospital stay (LOS) and its related factors after surgery in patients undergoing lobectomy for non-small cell lung cancer.

Methods: A descriptive retrospective study was conducted on 99 patients undergoing lobectomy for non-small cell lung cancer at University Medical Center Ho Chi Minh City. Data were extracted from a computerized database of patients who were hospitalized for lobectomy in the treatment of non-small cell lung cancer from January 2018 to December 2021. The primary outcome was the postoperative LOS.

Results: Median postoperative LOS was 5.2 days (interquartile range 4.8 to 6.8 days). The complication rate was 19.2%, of which Clavien-Dindo II accounted for the highest at 9.1%. The 30-day readmission rate was 13.1%. The median of LOS in the current cigarette smoker's group was 1.9 days higher than the never-cigarette smoker's group and 1.5 days higher than the former cigarette smokers (p<0.001). Tumor-nodes-metastasis (TNM) stage III showed the highest LOS compared to other stages (p=0.029). Open surgery and thoracoscopic conversion to open showed postoperative LOS about two days longer than thoracoscopic surgery (p<0.001). ​Performing muscle relaxation and early extubation, multimodal analgesia reduced postoperative LOS by 1.6 days (p<0.001), and preoperative physical therapy and early physical therapy at recovery reduced postoperative LOS by 1.3 days (p<0.001). There was a strong positive correlation between the duration of endotracheal retention, duration of thoracic drainage, amount of blood loss, and postoperative LOS (R>0.5, p<0.001). The duration of the Post-Anesthesia Care Unit and fasting time after surgery showed an average positive correlation with postoperative LOS (0.3<R<0.5, p<0.001). Preoperative fasting time had a weak positive correlation with postoperative LOS (R=0.265, p=0.008).

Conclusions: The median postoperative LOS was 5.2 days, and more than half of patients stayed in the hospital for over five days. Some factors affect the LOS, including current cigarette smokers, TNM stage, surgical approaches, some care processes such as early extubation, multimodal pain relief, physical therapy, vomiting, duration of thoracic drainage, amount of blood loss, duration of Post-Anesthesia Care Unit (hours), duration of thoracic drainage (days), preoperative and postoperative fasting time (hours). The study results help propose many changes in perioperative care for patients undergoing lung cancer surgery.

## Introduction

Lung cancer causes a large burden, with 1.8 million cases of mortality per year [1]. In a multimodal cancer treatment strategy, lobectomy is the preferred choice in patients with early-stage lung cancer [2]. However, major surgery such as lobectomy often has many potentially serious complications after surgery [3,4]; for patients who have undergone lobectomy, improving the quality of treatment to reduce the length of hospital stay (LOS), complications, and unexpected events remains a challenge for healthcare systems in many nations [5].

The Enhanced Recovery After Surgery Program (ERAS) program, an evidence-based, multidisciplinary perioperative care pathway, is designed to achieve early recovery after surgery by maintaining preoperative organ functions and reducing post-operative reactions [6-8]. ERAS has also been shown to be associated with the improvement of postoperative outcomes, including reduced LOS and the rate of pulmonary and cardiac complications after thoracic surgery [9,10]. The safety of ERAS has been demonstrated by a low rate of unexpected events without an impact on hospital readmission or perioperative mortality [11].

In 2019, the recommendations of the ERAS Association and the European Association of Thoracic Surgeons published the Guidelines for Enhanced Recovery After Lung Surgery. With strong evidence, ERAS is recommended for widespread use in major surgeries to reduce the LOS and limit complications and readmission rates [12,13]. Although the beneficial effects of ERAS have been proven, implementation at each medical facility is different. Due to the specific characteristics of each country's healthcare system, the resources of each hospital do not yet allow the effective application of ERAS in clinical practice. The application of ERAS in low-middle-income countries such as Vietnam still faces many challenges [14].

In Vietnam, the University Medical Center Ho Chi Minh City is one of the few hospitals qualified to apply ERAS in the treatment process [15]. However, the hospital's level of readiness to receive this program is unclear. It is necessary to conduct studies to evaluate the current status of post-operative recovery care according to conventional procedures (procedures without ERAS) in our center. This is an important step to identify the context and evaluate the impact of several factors to improve treatment results. This is the evidence to convince hospital leaders, surgeons, anesthesiologists, and medical staff to participate in ERAS completely, which may enhance the treatment effectiveness in lung cancer patients. This study is to determine the length of postoperative hospital stay and its related factors after surgery in patients undergoing lobectomy for non-small cell lung cancer.

## Materials and methods

Study settings and participants

A descriptive retrospective study was conducted on 99 patients who underwent lobectomy for non-small cell lung cancer at University Medical Center Ho Chi Minh City from January 2018 to December 2021. Inclusion criteria included (1) Patients diagnosed with lung cancer through pathological examination and (2) undergoing anatomical lung lobectomy. Exclusion criteria include (1) patients who do not comply with follow-up examinations after discharge; (2) patients who had surgery at University Medical Center Ho Chi Minh City but were transferred to another hospital for post-operative care after surgery.

Data collection and tools

Data were extracted from the hospital's computerized database of patients who were hospitalized for lobectomy in the treatment of non-small cell lung cancer from January 2018 to December 2021. Data extraction from the electronic medical record system is based on the International Classification of Diseases (ICD) code of the disease and the surgical method code.

The variables included demographic characteristics such as age group, sex, body mass index, cigarette smoking status, comorbidities, American Society of Anesthesiologists (ASA) classification, tumor location, tumor-node-metastasis (TNM) stage, lung cancer cell types, and cell differentiation. The group of treatment characteristics variables included operation method (open surgery, thoracoscopic surgery, thoracoscopic surgery convert to open), muscle relaxation and early extubation, multimodal pain relief, physical therapy before surgery, using opioids before surgery, physical therapy (before surgery, at recovery unit, after surgery), vomiting after surgery, amount of blood loss (mL), operation time (minutes), duration of endotracheal retention (hours), duration in the post-anesthesia care unit (hours), duration of thoracic drainage (days), preoperative fasting time (hours), postoperative fasting time (hours), urinary catheter retention time (hours), pain score.

The primary outcome was the postoperative LOS (day). Hospital discharge criteria include (1) pleural drainage tube removed; (2) no signs of infection; (3) pain control with oral analgesics; (4) x-ray shows good lung expansion, no significant effusion or air effusion and (5) able to oral feedings and did not require nasal cannula oxygen. Complications were defined depending on the Clavien-Dindo classification [16]. Re-admission was also a secondary outcome, determined by the readmission of the patients within 30 days.

Statistical analysis

The research data were entered using EpiData Entry software (version 3.1, EpiData Association) and analyzed using Stata software (version 16.0, Stata Statistical Software: Release 16, StataCorp LLC, College Station, TX, USA). T-test, One-way ANOVA, and Fisher's exact test were employed to measure associations with a significance level of p-value < 0.05 when the LOS were distributed normally. In the case of length of stay exhibiting an abnormal distribution, the Kruskal-Wallis test and the Spearman correlation were used to analyze. This study focuses on analyzing univariate associations between LOS and related factors.

## Results

Patient characteristics are shown in Table 1. The majority of participants were male (53.5%). There were 75 patients (75.8%) aged 60 or older. Half of the patients had a normal BMI (50.5%), and the overweight/obesity rate was 41.4%. ASA II classification accounted for the highest proportion, with 79.8%, followed by ASA III, with 16.2%. There were 62 never cigarette smokers (62.6%), 27 current cigarette smokers (27.3%), and ten former cigarette smokers (10.1%). Comorbidities such as hypertension accounted for the highest rate at 47.8%; in descending order were diabetes (16.2%) and coronary artery disease (9.1%).

**Table 1:** Characteristics of patients (n=99) BMI: Body mass index; ASA: American Society of Anesthesiologists; COPD: Chronic Obstructive Pulmonary Disease; TNM: Tumor-Node-Metastasis

Variables		Frequency	Proportion
Gender	Male	53	53.5
	Female	46	46.5
Age group	<60	24	24.2
	>=60	75	75.8
Living place	Urban area	35	35.4
	Rural area	64	64.6
BMI classification (BMI = weight in kilograms (kg) divided by the square of height in meters (m2))	Underweight (BMI < 18.5)	8	8.1
	Normal (BMI 18.5-22.9)	50	50.5
	Overweight (BMI ≥ 23)	41	41.4
ASA classification	I	4	4
	II	79	79.8
	III	16	16.2
Cigarette smoking status	Never cigarette smokers	62	62.6
	Former cigarette smokers	10	10.1
	Current cigarette smokers	27	27.3
Comorbidities	Hypertension	47	47.8
	Asthma /COPD	5	5.1
	Diabetes	16	16.2
	Dyslipidemia	2	2
	Coronary artery disease	9	9.1
	Heart failure	1	1
	Sequelae of stroke	1	1
	Thyroid dysfunction	3	3
	Anemia	2	2
	Other cancers	2	2
	Other diseases	13	13
Tumor location	Left-upper lobe	27	27.3
	Left-lower lobe	12	12.1
	Right-upper lobe	30	30.3
	Right-lower lobe	18	18.2
	Right-center lobe	12	12.1
TNM stage	IA	12	12.1
	IB	27	27.3
	IIA	25	25.2
	IIB	25	25.2
	IIIA	9	9.1
	IIIB	1	1.1
Lung cancer cell types	Adenocarcinoma	78	78.8
	Squamous cell carcinoma	20	20.2
	Large cell carcinoma	1	1.1
Cell differentiation	Well-differentiated	4	4
	Moderately differentiated	88	88.9
	Poorly differentiated	7	7.1

The highest recorded tumor location was in the right-upper lobe (30.3%), left-upper lobe (27.3%), and right-lower lobe (18.2%). Most patients had the second TNM stage with a total of 50.4%; the first TNM stage accounted for 39.4%, and the third TNM stage accounted for 10.2%. Regarding lung cancer cell types, 78.8% were adenocarcinoma; moderately differentiated accounted for the highest rate at 88.9% (Table 1).

Table 2 shows the characteristics of the treatment and care process. Sixty-eight patients underwent thoracoscopic surgery (68.7%). Ten patients assigned to thoracoscopic surgery were converted to open surgery, accounting for 10.1%. The rate of open surgery was 21.2% (21 patients).

**Table 2:** Characteristics of treatment and care process (n=99) (*) Median (interquartile range)

Variables		Frequency	Proportion
Operation method	Open surgery	21	21.2
	Thoracoscopic surgery	68	68.7
	Thoracoscopic surgery converts to open	10	10.1
Treatment and care process	Early endotracheal extubation (yes)	21	21.2
	Multimodal pain relief (yes)	55	55.6
	Physical therapy before surgery (yes)	32	32.3
	Using opioids before surgery (yes)	8	8.1
	Prophylactic antibiotics (yes)	99	100
	Physical therapy at the recovery unit (yes)	40	40.4
	Physical therapy after surgery at thoracic department (yes)	87	87.9
	Vomiting after surgery (yes)	15	15.2
	Amount of blood loss (ml)*	50 (30-150)	
	Operation time (minutes)*	170 (130-195)	
	Duration of endotracheal retention (hours)*	2 (1-4)	
	Duration in Post-Anesthesia Care Unit (hours)*	26 (21-40)	
	Duration of thoracic drainage (days)*	3 (3-5)	
	Preoperative fasting time (hours)*	13 (12-17)	
	Postoperative fasting time (hours)*	12 (10-16)	
	Urinary catheter retention time (hours)*	20 (17-24)	

There were 21 patients (21.2%) who received muscle relaxation and early extubation; 55 patients (55.6%) received multimodal pain relief; 32 patients (32.3%) received physical therapy before surgery; eight patients (8.1%) received opioids before surgery; 99 patients (100%) received prophylactic antibiotics; 40 patients (40.4%) received physical therapy at recovery unit; 87 patients (87.9%) were performed physical therapy after surgery in thoracic department. The data showed that 15 cases of postoperative vomiting accounted for 15.2% (Table 2).

Blood loss had a median of 50 mL (quartile of 30-150 mL). The median of these variables was reported in turn: operation time (170 minutes), duration of endotracheal retention (two hours), duration in the post-anesthesia care unit (26 hours), duration of thoracic drainage (three days), preoperative fasting time (13 hours), postoperative fasting time (12 hours), urinary catheter retention time (20 hours) (Table 2).

Table 3 shows the LOS and clinical outcomes. The median length of postoperative hospital stay was 5.2 days (interquartile range 4.8 to 6.8 days). The median reported total hospital stay was 12.2 days (interquartile range 9.2 to 16.4 days). The complication rate was 19.2%, of which Clavien-Dindo II accounted for the highest at 9.1%. There were two cases of re-operation, accounting for 2%. The 30-day readmission rate was 13.1% (13 patients), including 10 patients with pneumonia.

**Table 3:** Length of postoperative hospital stay and clinical outcomes (n=99) LOS: length of hospital stay

Variables		Frequency	Proportion
Postoperative LOS	≤ 72h	41	41.4
	> 72h	58	58.6
Complications	No complication	80	80.8
	Clavien- Dindo I	4	4.1
	Clavien- Dindo II	9	9.1
	Clavien- Dindo IIIa	4	4
	Clavien- Dindo IIIb	2	2
Re-operation	Yes	2	2
	No	97	98
Re-admission to hospital in 30 days	Yes	13	13.1
	No	86	86.9
Reasons for re-admission (n=13)	Pneumonia	10	76.9
	Haemoptysis	1	7.7
	Stroke	1	7.7
	Respiratory failure	1	7.7

Table 4 shows the relation between LOS and population characteristics. There was an association between LOS and cigarette smoking status, coronary heart disease, and the stage of cancer. Specifically, the median of LOS in the current cigarette smoker's group was 1.9 days higher than the never cigarette smokers group and 1.5 days higher than the former cigarette smokers (p<0.001). Those with coronary artery disease had a median LOS of 6.9 days, 1.7 days higher than those without coronary artery disease (p=0.009). TNM stage III showed the highest LOS compared to other stages (p=0.029).

**Table 4:** Relation between postoperative hospital stay and patients' characteristics (n=99) (#) Kruskal-Wallis test (*) Median (Interquartile range) BMI: Body mass index; ASA: American Society of Anesthesiologists; COPD: Chronic Obstructive Pulmonary Disease; TNM: Tumor-Node-Metastasis

Variables		Postoperative Length of hospital Stay*	P-value^#^
				Gender	Male	5.6 (4.8-6.9)	0.076
Female	5.1 (4.5-6.2)						
Age group	<60	5.2 (4.6-6.9)	0.578
	>=60	5.2 (4.8-6.8)	
BMI classification	< 18.5	5.1 (4.3-10.9)	0.855
	18.5-22.9	5.7 (4.8-6.8)	
	≥ 23	5.1 (4.8-6.7)	
ASA classification	I	5.5 (4.1-6.9)	0.128
	II	5.2 (4.8-6.7)	
	III	6.9 (4.9-7.9)	
Cigarette smoking status	Never cigarette smokers	5 (4.3-5.8)	<0.001
	Former cigerette smokers	5.4 (4.8-6)	
	Current cigarette smokers	6.9 (5.9-8.9)	
Hypertension	Yes	5.2 (4.9-7.0)	0.09
	No	5.1 (4.6-6.8)	
Asthma /COPD	Yes	7.0 (5.5-7.8)	0.171
	No	5.2 (4.8-6.8)	
Diabetes	Yes	5.0 (4.8-5.7)	0.291
	No	5.2 (4.8-6.8)	
Dyslipidemia	Yes	5.5 (5.1-5.8)	0.97
	No	5.2 (4.8-6.8)	
Coronary artery disease	Yes	6.9 (5.5-14.0)	0.009
	No	5.2 (4.8-6.8)	
Thyroid dysfunction	Yes	4.7 (4.2-5.0)	0.123
	No	5.2 (4.8-6.8)	
Anemia	Yes	6.7 (5.0-7.8)	0.385
	No	5.2 (4.8-6.8)	
Other cancers	Yes	4.4 (3.9-4.9)	0.117
	No	5.2 (4.8-6.8)	
Tumor location	Left-upper lobe	5. 9(4.8-6.9)	0.86
	Left-lower lobe	5.2 (4.2-6.0)	
	Right-upper lobe	5.2 (4.9-6.8)	
	Right-lower lobe	5.0 (4.8-7.0)	
	Right-center lobe	5.1 (4.8-7.0)	
TNM stage	IA	4.8 (4.1-5.5)	0.029
	IB	5.1 (4.7-6.8)	
	IIA	5.2 (4.9-6.8)	
	IIB	6.1 (5.0-6.9)	
	IIIA	5.2 (4.9-6.8)	
	IIIB	4.5 (4.5-4.5)	
Lung cancer cell types	Adenocarcinoma	5.2 (4.8-6.9)	0.692
	Squamous cell carcinoma	5.2 (4.8-6.8)	
	Large cell carcinoma	4.8 (4.8-4.8)	
Cell differentiation	Well-differentiated	5.1 (5.0-5.5)	0.075
	Moderately differentiated	5.2 (4.8-6.8)	
	Poorly differentiated	6.9 (5.6-14.0)	

Table 5 gives information about the relationship between LOS and treatment characteristics. There was a statistically significant difference in LOS between surgical methods. Open surgery and thoracoscopic conversion to open showed LOS about two days longer than thoracoscopic surgery (p<0.001). ​Performing muscle relaxation and early extubation, multimodal analgesia reduced LOS by 1.6 days (p<0.001), and preoperative physical therapy and early physical therapy at recovery reduced LOS by 1.3 days (p<0.001). In addition, it was noted that people who had vomiting after surgery had nearly one day longer in the hospital than people who did not have (p<0.001). The median of LOS in the uncomplicated group was 2.9 days lower than the group with complications (p<0.001). There was a strong positive correlation between the duration of endotracheal retention, duration of thoracic drainage, amount of blood loss, and LOS (R>0.5, p<0.001). The duration of the post-anesthesia care unit and fasting time after surgery showed an average positive correlation with LOS (0.3<R<0.5, p<0.001). Preoperative fasting time had a weak positive correlation with LOS (R=0.265, p=0.008).

**Table 5:** Relation between postoperative length of hospital stay and treatment characteristics (*): Median (Interquartile range) (#): Two-sample Wilcoxon rank-sum test (##): Kruskal-Wallis test (a): Spearman correlation

Variables		Postoperative Length of hospital Stay *	P-value
Operation method	Open surgery	6.9 (5.9-8)	<0.001^##^
	Thoracoscopic surgery	5 (4.6-5.8)	
	Thoracoscopic surgery converts to open	6.9 (5.8-7.9)	
Early extubation	Yes	4.3 (4-4.8)	<0.001^#^
	No	5.9 (5-6.9)	
Multimodal pain relief	Yes	4.9 (4.2-5.8)	<0.001^#^
	No	6.5 (5.2-8)	
Physical therapy before surgery	Yes	4.7 (4.1-4.9)	<0.001^#^
	No	6 (5.2-7)	
Using opioids before surgery	Yes	5.1 (4.9-8.8)	0.612^#^
	No	5.2 (4.8-6.8)	
Physical therapy at the recovery unit	Yes	4. 9 (4.2-5.2)	<0.001^#^
	No	6.2 (5-7.9)	
Physical therapy after surgery	Yes	5.2 (4.8-6.8)	0.577^#^
	No	5.6 (4.9-6.9)	
Vomiting after surgery	Yes	6 (5.2-14)	0.005^#^
	No	5.1 (4.8-6.8)	
Complication	Yes	8 (6,8-14)	<0,001^#^
	No	5,1 (4,7-5,9)	
Amount of blood loss (ml)		R=0.526	<0.001^a^
Operation time (minutes)		R=0.064	0.529^a^
Duration of endotracheal retention (hours)		R=0.748	<0.001^a^
Duration of Post-Anesthesia Care Unit (hours)		R=0.476	<0.001^a^
Duration of thoracic drainage (days)		R=0.678	<0.001^a^
Preoperative fasting time (hours)		R=0.265	0.008^a^
Postoperative fasting time (hours)		R=0.358	<0.001^a^
Urinary catheter retention time (hours)		R=0.068	0.504^a^

## Discussion

This was a retrospective study conducted on 99 patients undergoing lobectomy to treat non-small cell lung cancer to investigate the LOS and factors related to the LOS after surgery. The study also further determined the complication rate, hospital readmission rate, and associated factors. The results of this study are intended to provide information to develop and implement the ERAS program application at our hospital.

LOS is a substantial indicator to assess the efficiency of healthcare service management and patient’s quality of care. Perioperative care should be designed to minimize the impacts of surgery, allowing the patient to return to their normal daily life as soon as possible. LOS is a commonly used measure of recovery after surgery [17,18]. The prolonged hospital stays increase medical fees and cause hospital overload. Prolonged hospital stays have been associated with risks of hospital infections and reduce the quality of life of patients [19]. Although the criteria for hospital discharge at each center are different, most of the criteria revolve around the patient's complete recovery, no serious complications, stable vital signs, and the ability to perform self-care and pain control with oral medications. That is why we use LOS as the main indicator to investigate patient care status after lobectomy, thereby making reasonable interventions when applying the ERAS program.

Since the period before 2021, during which time we have not applied ERAS in lung surgery, we have focused on applying minimally invasive surgical methods to reduce hospital stays [20]. Median hospital stay in our study was 5.2 days (interquartile range 4.8 to 6.8 days), similar to María Teresa Gómez-Hernández (overall mean LOS was 5.53±3.82 days). However, Gómez-Hernández’s study, when analyzed by period, shows that the LOS gradually decreased, from six days (IQR: two days) between 2011 and 2014 to five days (IQR: two days) from 2015 to 2018 and three days (IQR: two days) during the period from 2019 to 2021 [21]. This shows we need to improve more because more than half of patients stay in hospital for over five days (58.6%). It can be seen that not only the application of minimally invasive surgical methods can reduce hospital stays, but other factors also influence it.

Many factors are related to a patient's hospital stay, including individual patient factors. Because health system resources are limited, knowing these factors can help managers make decisions to optimize the use of resources. Omid Khosravizadeh's research results show that many factors, such as age, employment, marital status, previous hospitalization history, patient's condition upon discharge, payment method, and type of treatment, may affect the length of stay [22]. According to Cameron DW, advanced age and a greater number of comorbidities were associated with prolonged hospitalization [23]. A study on 3,473 lobectomy patients showed that age, BMI, male sex, FEV1, and DLCO were factors predicting prolonged hospital stay after lung resection surgery [24]. Our study found the factors related to the LOS after surgery, such as cigarette smoking status, coronary artery disease, and TNM stage. This is also the information for us to implement the application of the ERAS program, including planning to counsel patients to quit smoking and stabilize underlying diseases before performing surgery. Detecting the disease early also makes the surgery go more smoothly. This emphasizes regular health checks and early cancer screening, a problem faced in low-income countries with limited access to healthcare.

The patient's care and treatment process before, during, and after surgery significantly affects the hospital stay. Our study records many factors in the treatment and care process related to the postoperative hospital stay, the complication rate, and the re-hospitalization rate, including surgical method, early extubation, multimodal analgesia, and exercise. Postoperative physical therapy, vomiting, blood loss, intubation time, pleural drainage time, fasting time before and after surgery, and time in the recovery room. This may indicate that the surgical approach and perioperative care aim to limit the impact of surgery and promote optimal recovery after surgery, allowing the patient to return home and continue. Continue daily activities as safely and quickly as possible. Overall improvement of the entire care and surgical process has been proven to be more effective in safely shortening hospital stays than focusing on a certain step (for example, focusing on developing less invasive techniques but forgetting that pain relief methods and post-operative nursing care are also affected). The latest guidelines on ERAS process development largely substantiate this [12,25].

The strength of this study is the relatively large sample for a single-center study, standardized surgical procedure, and comprehensiveness of variables studied. This is also a study on factors related to hospital stay before intervention in the ERAS program to help managers have a more comprehensive view of implementation. The limitation of this study is quite clear: it is only a single-center study. This study only reports single-center results, so the ability to generalize to other medical facilities is also a limitation because the actual situation in each hospital is different. The other limitations related to the retrospective study design include the potential for missing data, selection bias, and lack of control over the variables measured. Confounding factors about background characteristics are bias factors. During the retrospective data collection process, information collected from medical records may deviate from primary data.

Research also shows that developing comprehensive care is necessary to improve the quality of recovery after surgery and help early discharge from the hospital. This finding emphasizes that short LOS results from a good perioperative care program and should not be considered a goal in itself.

## Conclusions

The median LOS was 5.2 days, and more than half of the patients stayed in the hospital for longer than five days. Some factors affect the LOS, including current cigarette smokers, TNM stage, surgical approaches, some care processes such as early extubation, multimodal pain relief, physical therapy, vomiting, duration of thoracic drainage, amount of blood loss, duration of post-anesthesia care unit (hours), duration of thoracic drainage (days), preoperative and postoperative fasting time (hours). The study results help propose many changes in the clinical practice of perioperative care for patients undergoing lung cancer surgery in the University Medical Center Ho Chi Minh City.
